# Optimal patient protocols in regional acute stroke care

**DOI:** 10.1007/s10729-020-09524-2

**Published:** 2021-02-23

**Authors:** B. L. Garcia, R. Bekker, R. D. van der Mei, N. H. Chavannes, N. D. Kruyt

**Affiliations:** 1 Public Health and Primary Care, Leiden University Medical Center, Leiden, Netherlands; 2 Department of Mathematics, Vrije Universiteit Amsterdam, Amsterdam, Netherlands; 3 Stochastics Group, CWI, Amsterdam, Netherlands; 4 Neurology Department, Leiden University Medical Center, Leiden, Netherlands; 5 University NeuroVascular Center (UNVC), Leiden-The Hague, The Netherlands

**Keywords:** Operations research, Facility location, Allocation protocol, Mothership, Drip-and-ship, Mixed integer linear programming, Volume-dependent in-hospital delays

## Abstract

In acute stroke care two proven reperfusion treatments exist: (1) a blood thinner and (2) an interventional procedure. The interventional procedure can only be given in a stroke centre with specialized facilities. Rapid initiation of either is key to improving the functional outcome (often emphasized by the common phrase in acute stroke care “time=brain”). Delays between the moment the ambulance is called and the initiation of one or both reperfusion treatment(s) should therefore be as short as possible. The speed of the process strongly depends on five factors: patient location, regional patient allocation by emergency medical services (EMS), travel times of EMS, treatment locations, and in-hospital delays. Regional patient allocation by EMS and treatment locations are sub-optimally configured in daily practice. Our aim is to construct a mathematical model for the joint decision of treatment locations and allocation of acute stroke patients in a region, such that the time until treatment is minimized. We describe acute stroke care as a multi-flow two-level hierarchical facility location problem and the model is formulated as a mixed integer linear program. The objective of the model is the minimization of the total time until treatment in a region and it incorporates volume-dependent in-hospital delays. The resulting model is used to gain insight in the performance of practically oriented patient allocation protocols, used by EMS. We observe that the protocol of directly driving to the nearest stroke centre with special facilities (i.e., the mothership protocol) performs closest to optimal, with an average total time delay that is 3.9% above optimal. Driving to the nearest regional stroke centre (i.e., the drip-and-ship protocol) is on average 8.6% worse than optimal. However, drip-and-ship performs better than the mothership protocol in rural areas and when a small fraction of the population (at most 30%) requires the second procedure, assuming sufficient patient volumes per stroke centre. In the experiments, the time until treatment using the optimal model is reduced by at most 18.9 minutes per treated patient. In economical terms, assuming 150 interventional procedures per year, the value of medical intervention in acute stroke can be improved upon up to € 1,800,000 per year.

## Highlights

- Regional acute stroke care with two reperfusion treatments is modeled using a mathematical optimization model.
- The time until treatment is minimized based on the joint decisions of treatment locations and the allocation of acute stroke patients.
- The optimization model incorporates the impact of patient volume on in-hospital time delays.
- Experiments with six regions in the Netherlands showed that mothership is in most cases preferred over drip-and-ship and time until treatment can be reduced by at most 18.9 minutes per treated patient.

## Introduction

Fast treatment of acute stroke is paramount and increases the prospects of good clinical outcome [1–8]. The passing of time is the most pivotal factor limiting clinical efficacy. For example, for every 15 minute delay to treatment, the chances of independent ambulation substantially decreases and 1 month of disability-free life is lost [9]. Two options are available to treat acute stroke, depending on patient characteristics. Both treatments aim to re-establish blood flow to the brain. The first is intravenous thrombolysis treatment (IVT) for which the majority of patients are eligible, but IVT is only effective within 4.5 hours from onset of stroke. The second is intra-arterial thrombectomy (IAT), which is a relatively new treatment option only applicable to a subset of selected ischemic stroke patients (4.5% according to Dutch Acute Stroke Audit), but can be applied up to 24 hours after symptom start. Importantly, in clinical practice, IAT is administered on top of IVT in approximately 21% of the IVT treated patients, but this percentage is increasing [19]. Moreover, of IAT treated patients, over 90% were first treated with IVT [25].

Whereas IVT is a treatment option available in most hospitals, so-called primary stroke centres (PSC’s), IAT is only available in so-called comprehensive stroke centres (CSC’s). In general, most regions possess a plurality of PSCs, but only a few CSCs, since the latter requires elaborate treatment techniques and specialized personnel. Patient allocation by emergency medical services (EMS) would ideally be based on IAT eligibility, but this requires in-hospital assessment. This means IAT eligibility can only be assessed at a centre and not at the patient location. It is therefore key to determine appropriate CSC locations on the regional level (i.e., decide which stroke centres are going to administer IAT for their area).

The following five factors influence the regional time delays in acute stroke care: (1) patient location; (2) regional patient allocation (i.e., regional allocation protocol prescribing to which type of stroke centre a suspected stroke patient is allocated by the EMS); (3) travel times of EMS; (4) PSC and CSC locations; and (5) in-hospital delays. It is not possible to influence the patient location, but the other factors can be controlled to some extent. The focus of this paper is on the second and fourth factor, respectively, the patient allocation protocol and the PSC/CSC locations. These two factors also influence the other two controllable factors, namely the EMS travel times and in-hospital delays. Increased patient load in a stroke centre improves in-hospital logistics and typically leads to shorter delays in stroke care [14, 29–31]. In-hospital delays can thus be controlled to an extent through the clever allocation of patients. Currently, unsubstantiated decisions regarding regional patient allocation and PSC and CSC locations are prevalent. A model-based method of organizing acute stroke care therefore offers great opportunities for reducing time delays until start treatment.

In clinical practice, two different patient allocation protocols are in use: *drip-and-ship* and *mothership* [17, 18, 28]. In the drip-and-ship protocol, suspected stroke patients are always allocated to the nearest stroke centre (either PSC or CSC) in order to initiate IVT as soon as possible. If a patient admitted to a PSC appears IAT eligible, subsequent allocation to a CSC follows. In general, this protocol results in short delays before IVT, but longer delays to IAT due to inter-hospital transportation. In the mothership protocol, suspected stroke patients are allocated directly to a CSC, often bypassing a PSC. In general, this protocol results in short delays before IAT, but relatively longer delays before IVT due to longer transportation times between patient location and CSC, compared to the shorter allocation time to a PSC. Essentially, neither protocol is optimal for a given region. The optimal protocol for a certain region may show elements of both the mothership and the drip-and-ship protocols, but may also use another allocation (e.g. allocate some demand to a PSC on the route to a CSC). The optimal allocation follows from the optimization model as introduced in Section 4.

Next to the allocation protocol, a strategic decision is the determination of which stroke centre should only administer IVT (i.e. become a PSC) and which should also administer IAT (i.e. become a CSC). To guarantee a certain degree of expertise at a CSC, a lower bound on the number of acute stroke patients offered at a location is required. In addition, the volume of acute stroke patients affects in-hospital delay [14, 29–31]. As IAT becomes a more familiar treatment option, this is an opportune moment to make well-founded strategic location decisions for PSCs and CSCs.

In the current paper, we propose a mathematical optimization model upon which to base the allocation protocol and PSC and CSC locations to minimize regional time delays, taking the impact of patient volume on in-hospital delays into account. The model is formulated as a mixed integer linear program (MILP) and solved using CPLEX. We also compare our optimized patient allocation protocol with drip-and-ship and mothership protocols, providing insight in the differences in performance. This research focuses on reducing delays to treatment initiation for the patient.

We see that the relative performance of mothership and drip-and-ship strongly depends on the region (population densities, stroke centre locations) as well as treatment-related parameters. As a rule of thumb, we observe the following: mothership performs well in urban areas and in regions where the CSCs are centrally located. Also, we see that mothership typically shows near-optimal performance when the fraction of IAT eligibility exceeds 50%. Vice versa, drip-and-ship performs well in rural areas and fractions of IAT eligibility of 30% or smaller, assuming that the patient volume per stroke centre is sufficiently large.

There are two related areas of existing literature. The first area stems from the clinical domain for strokes. For instance, [16, 20] assess eligibility of IAT through clinical trials. More closely related are [15] and [17]. The paper [15] considers some scenarios for patient allocation based on clinical data, whereas [17] analyzes the difference between the drip-and-ship and mothership protocols based on current locations and allocation characteristics.

The second area of related work concerns the area of facility locations problems. There is a long history on location problems and a sizeable amount of literature by now. We refer to the book [40] for an overview of location problems in general, including some optimization models. The book contains a chapter dedicated to location problems in health care [39]; we also refer to [12] for a survey on health care facility location problems. In the drip-and-ship protocol, patient are allocated to the nearest stroke centre. The studies [41, 42] also consider health care settings that incorporate closest assignment constraints in the optimization model.

Another branch of literature takes congestion into account for facility location problems and the allocation of clients to resources. The allocation prescribes the arrival rates at locations, giving rise to queueing phenomena [43]. Such models lead to non-linear optimization models, which are optimized using heuristics in [44, 45]. In [46, 47] the goal is to minimize travel times plus waiting times in a health care network, where the waiting times follow from queueing expressions; the authors exploit an MILP combined with piece-wise linear approximations to linearize the goal function and all constraints.

Due to the two types of treatments and centres, the problem falls in the class of multi-flow two-level hierarchical facility location problems [10, 11]. An example of a two-level hierarchical model with a proportion of patients being transferred between health centres and hospitals is [48]; the optimization model is solved using five heuristic procedures. In [49] an MILP model is used for location-allocation problems with district and central hospitals. The design of a long-term multi-facility care network using large scale MILP’s is considered in [34, 51]. Also of interest is [50], which considers a location-allocation problem that aims to optimize health gains directly using an MILP.

Even though many researches modelled healthcare networks using MILP, our understanding is that there are no studies that incorporate acute patients in a chain of stroke centres and volume-dependent delays in the facility location literature.

Our contribution is two-fold. First, we formulate optimization models for locations and allocation protocols for treatment of acute stroke in the spirit of two-level facility location models. Specifically, we incorporate volume-dependent in-hospital delays and practically common protocols (drip-and-ship and mothership). Second, we provide insight in the performance of the practically oriented protocols. The optimization framework is generic, but we imagine that the practical heuristics may be preferred in practice.

The paper is organized as follows. In Section 2 we give some background and introduce notation for the modeling of stroke care. Some initial insights for the different protocols is presented in Section 3 based on simulation results of a stylized example. The optimization formulation is given in Section 4. Numerical experiments can be found in Section 5, whereas Section 6 concludes.

## Modeling regional acute stroke care logistics

**Fig. 1:**
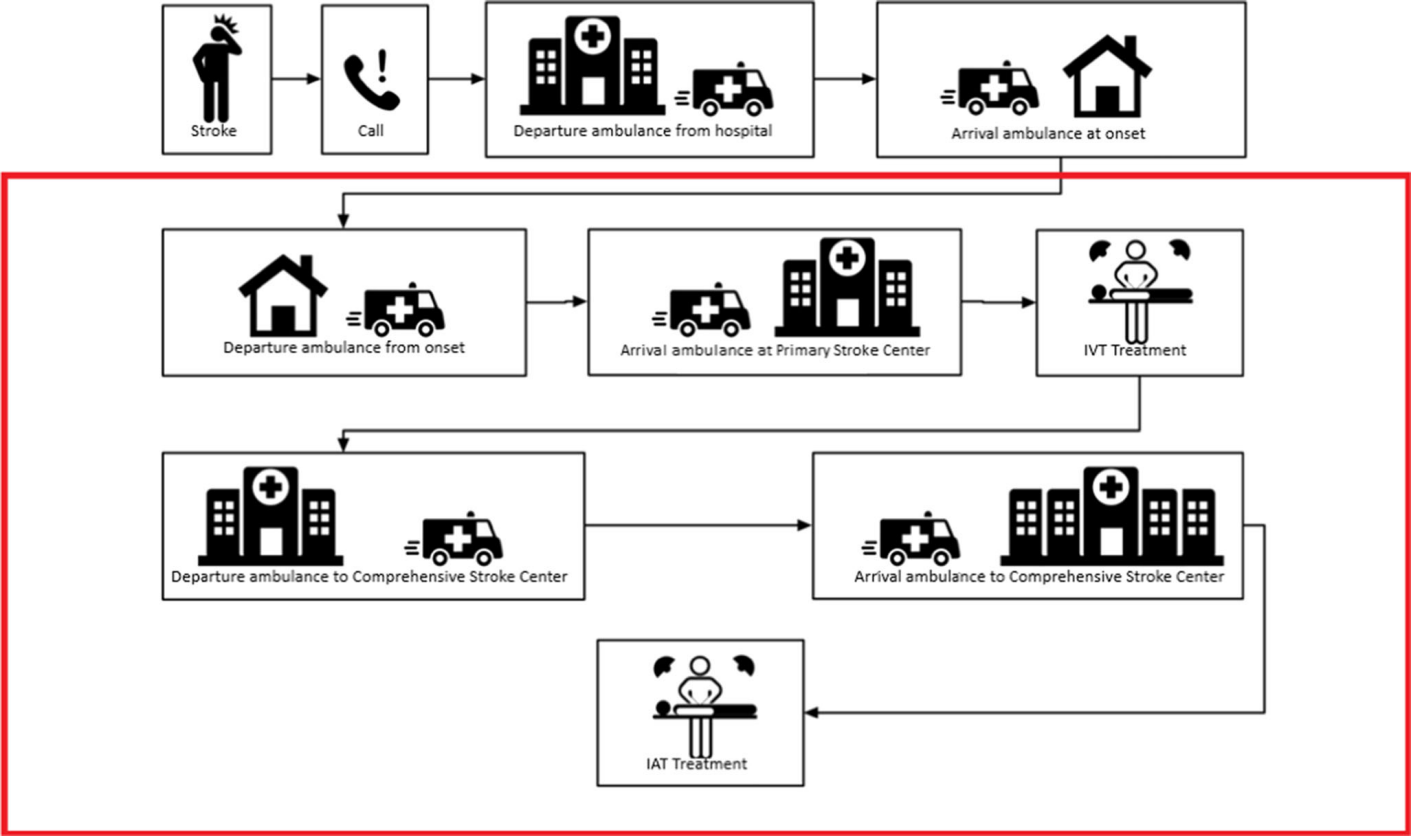
Visualization of the patient flow for acute stroke; we focus on the boxed area

In this section, we provide some background about acute stroke care and introduce notation for the model. In Fig. 1, the possible patient flows from onset to start treatment are depicted. We focus on the flow starting from departure ambulance from patient location and ending with the start of either IVT or IAT, as depicted by the red area in Fig. 1. We call this time the SDST (Scene-Departure-to-Start-Treatment). Thus, the time from onset until the ambulance arrives at the scene is left out of scope; this duration depends on many external factors and is not affected by patient allocation protocols and PSC and CSC locations. Below, we introduce the elements of the model.

### Location

A region is assumed to consist of a finite number of non-overlapping areas, called locations. Typically, a location corresponds to a postal code area (as in e.g. [13]). The non-overlapping areas (or locations) are relatively small such that the distance within a location may be assumed to be zero. We assume that demand may occur from each location, whereas there is a smaller set of treatment locations of stroke centres.

### Demand

For the demand we consider all IVT- and IAT-eligible patients. The suspected stroke patients that do not require treatment are not affected by the allocation protocol and the PSC and CSC locations. Therefore, this group is considered to be out of scope. Note that this group does affect the occupation of EMS to some extent. We denote the demand per year at demand location *i* by *w*_*i*_, and let $P = {\sum }_{i} w_{i}$ be the total demand in the region per year. Typically, the demand per location depends on demographic characteristics and can be retrieved from open sources.

### PSC and CSC locations

The model allocates treatments (IVT and/or IAT) to the potential treatment locations. These typically correspond to existing stroke centres and are a subset of all locations in a region. Treatment locations may correspond to the locations of demand of IVT- and IAT-eligble patients, but may also be considered as separate locations.

### Travel time

We denote the travel time between locations *i* and *j* by *d*_*ij*_. In this study, travel times are based on average emergency travel time estimates between two locations provided by the Dutch National Institute for Public Health and the Environment (RIVM). The model can also be used with travel times from applications (e.g. Google Maps) or distance measures; for the latter, we refer to [21] for a more elaborate discussion.

### Eligibility of IAT treatment

The probability of being IAT eligible, denoted by *P*(*IAT*), depends on individual patient characteristics [22], but not on location or transportation. In this model, *P*(*IAT*) is constant over time and is defined as the fraction of IVT patients that are eligible to receive IAT.

### In-hospital delays

The in-hospital delay is the time from arrival at the door of the stroke centre until the start of the specific treatment (IVT or IAT). In-hospital delays inversely depend on the volume of IVT treated patients with higher volumes leading to shorter delays [14, 29–31]; larger patient volumes provide experience and often also lead to organizational improvements. In addition, very small patient volumes are undesirable; a sufficient volume of patients should be treated to ensure that skills are maintained and efficiency is not becoming an issue [32]. Hence, we assume that in-hospital delays behave as a convex function of the patient volume.

### Minimum volume requirement for IVT/IAT

To maintain a sufficient level of expertise in the treatment of acute stroke, it is crucial to treat a minimum number of patients. In clinical practice, there is a minimal requirement for the volume of IVT/IAT patients [26]. In our model, the minimal number of IVT and IAT patients per year are denoted by *r*_*IVT*_ and *r*_*IAT*_, respectively.

## Insights from a stylized example

In this section, we investigate the impact of the different allocation protocols that are currently in use by considering a stylized example. The primary goal is to obtain insight in how the allocation protocols - mothership, drip-and-ship, and optimal - behave for different values of *p* := *P*(*IAT*) and the number of PSC facilities relative to the number of CSCs. We assume that the region of interest is a square and denote this square by *U*; without loss of generality we assume *U* to be the unit square. There is a single CSC and there are *n* PSCs, which are uniformly distributed over *U*. The demand point is also uniformly distributed over *U*. The distance *d*_*ij*_ between two points *i* and *j* is given by the Euclidean distance (the *L*^2^ norm); see Remark 3 for the Manhattan distance.

Note that if the location of facilities are given and in-hospital delays are independent of the number of patients treated at the facilities (and there are no upper and lower bounds), the optimal allocation protocol can easily be obtained. This is due to the fact that the optimization can be separately carried out for each demand point. The optimal allocation for demand point *i* is then determined by

1 $$ \underset{{ k=0,\ldots,n}}{arg~{min}} \left\{ d_{i j_{k}} + b_{k, IVT} + p (d_{j_{k} j_{0}} + b_{IAT}) \right\}, $$

where *j*_0_ is the CSC location and *j*_*k*_ is the location of PSC *k*, *k* = 1,…,*n* (with $d_{j_{0}, j_{0}} =0$). Here, *b*_*k*, *i*_ denotes the in-hospital delay for treatment *i* ∈{*IV* *T*, *IAT*} at location *k*. For mothership, the SDST is $d_{i j_{0}} + b_{0, IVT} + p b_{IAT}$, whereas for drip-and-ship the SDST is $\min \limits _{k=0,\ldots ,n} \left \{ d_{i j_{k}} \right \} + b_{k^{*}, IVT} + p (d_{j_{k^{*}} j_{0}} + b_{IAT})$, where $k^{*} = arg min_{k=0,\ldots ,n} d_{i j_{k}}$. In this example, we focus on the travel time and exclude in-hospital delays (that is, assume them to be identical).

**Fig. 2:**
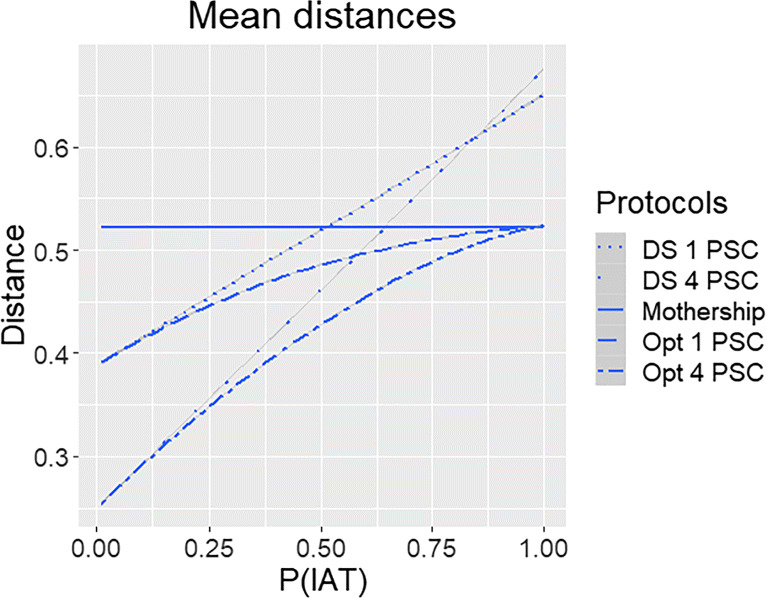
Mean of the travel distances for various *p* = *P*(*IAT*) and allocation protocols

In Fig. 2, the mean of the travel distances are shown for the three different protocols and for *n* = 1 and *n* = 4 based on 10,000 simulations per instance. We varied *p* to see the impact of the three protocols. Naturally, the mothership protocol does not depend on *p* and *n*. The performance (generally) improves with 4 PSCs compared to the situation with 1 PSC, which may be evidently explained by a smaller distance to the nearest facility and more options for routing patients in case of the optimal protocol. Moreover, the optimal protocol clearly outperforms the other two protocols (for the same number of PSCs). When *p* *↓* 0 drip-and-ship is optimal as there is no need for further allocation of patients to a CSC. On the other hand, for *p* *↑* 1, mothership is optimal, as all patients should eventually be allocated to a CSC. For *p* = 0.5 the relative gain in mean travel distance of the optimal protocol compared to mothership is roughly 7% and 18% for *n* = 1 and *n* = 4, respectively.

Comparing mothership with drip-and-ship, we see that drip-and-ship performs better for *p* smaller than 0.5 in case *n* = 1, where roughly *p* = 0.5 is the break-even point. For *n* = 4, the break-even point shifts to a slightly larger value of *p*. Interestingly, when *p* is above 0.8 the drip-and-ship protocol with *n* = 1 has a smaller mean distance than for the case that *n* = 4. This is due to the fact that it is more likely that the CSC is the nearest location in case *n* = 1 than for *n* = 4.

The example above provides some fundamental insight in how *P*(*IAT*) and the number of PSCs affect the allocation protocols. Moreover, Equation (1) provides a simple heuristic for determining allocation strategies. With volume-dependent IVT treatment times and different demographic regions, the patterns described in this section will roughly remain valid, but the performance loss may shift. In Section 5, we elaborate on this when demographic regions are taken into account in an optimization model.

### *Remark 1*

Distances between random points have been extensively studied in the literature on spatial point processes. For instance, the average distance between two random points in the unit square is

$\left (2+\sqrt {2}+5(\ln (1+\sqrt {2})\right )/15 \approx 0.5214$, see p. 171 of [23], corresponding to the simulation results for mothership. We also refer to [23, 24, 27] for further examples. Observe, however, that the drip-and-ship and optimal allocation protocols are considerably more intricate to derive analytically.

### *Remark 2*

It may be easily verified that there is a *p*^∗^ such that drip-and-ship outperforms mothership for *p* < *p*^∗^, and vice versa for *p* > *p*^∗^. This follows from the intermediate value theorem, the fact that the performance of drip-and-ship is decreasing in *p* (and independent of *p* for mothership), and the boundary cases *p* = 0 and *p* = 1.

### *Remark 3*

Next to the Euclidean distance, another common distance metric is the Manhattan distance (*L*^1^ norm). The distance between points (*x*_1_,*y*_1_) and (*x*_2_,*y*_2_) is then |*x*_1_ − *x*_2_| + |*y*_1_ − *y*_2_|. Although the actual distances for the *L*^1^ norm differ from those of the *L*^2^ norm, the relative results are very similar and are thus not presented here.

## Optimization framework

In this section, we propose an optimization model for jointly determining the allocation protocols and PSC and CSC locations, in order to minimize the mean SDST. The model is formulated as an MILP based on a modification of a multi-flow two-level hierarchical facility location problem [11]. A primary feature that we include is that in-hospital delays depend on patient volumes. In addition to the optimal allocation protocol, we also discuss the model for optimal locations in case drip-and-ship is enforced.

### Parameters and variables

An outline of regional stroke care, including notation, has been given in Section 2. As mentioned, we assume that there is a minimum requirement *r*_*c*_ for patients treated of type *c* ∈{*IV* *T*, *IAT*} at open stroke centres. Especially because IAT is still relatively new, we also include a maximum number of PSC and/or CSC locations in the region, denoted by *p*_*c*_. The binary decision variable *h*_*jc*_ equals 1 if stroke centre at location *j* ∈ *J* provides treatment type *c* ∈ *C*, and 0 otherwise.

The allocation of patients from their demand location to the PSC is given by the binary decision variables *y*_*ij*_ (*i* ∈ *I*, *j* ∈ *J*), where *y*_*ij*_ takes value 1 if a patient from demand point *i* is allocated to stroke centre location *j*, and 0 otherwise. Subsequently, the potential allocation of patients from a PSC to a CSC is determined by *v*_*ij*_, which equals the total number of patients from PSC location *i* ∈ *J* to CSC location *j* ∈ *J*. These locations can be identical in case a stroke centre provides both IVT and IAT. The distance *d*_*jj*_ (*j* ∈ *J*), may take negative values to model the situation where there is an efficiency gain in delays when the patient remains in the same stroke centre. Thus, *d*_*ij*_ ≥ 0 for (*i* ∈ *I* ∪ *J*, *j* ∈ *J*) and *j*≠*i*, whereas $d_{jj} \in \mathbb {R}$.

**Fig. 3:**
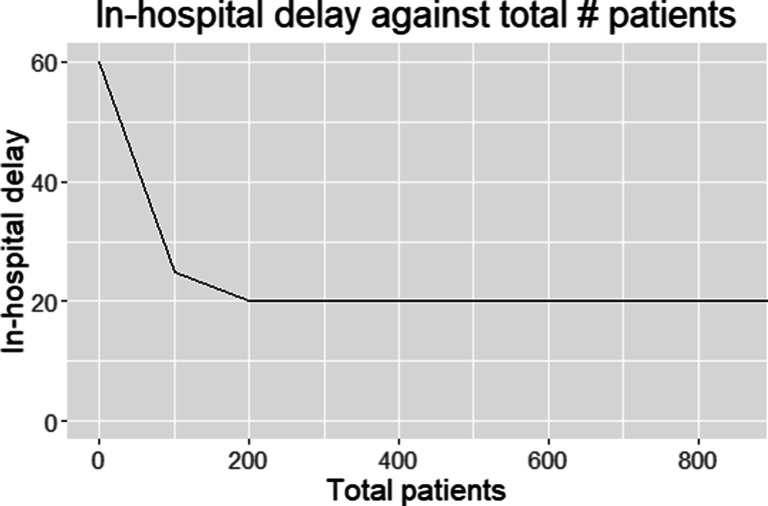
Example of the piece-wise linear approximation of the in-hospital delay as a function of volume of IVT treatments

We assume that in-hospital delays are stroke centre specific, i.e., let *b*_*j*, *c*_ be the in-hospital delay for location *j* and treatment of type *c*. As a relation between the number of IAT patients and in-hospital delays has not yet been established, we assume this delay to be independent of the volume. From [14], it follows that in-hospital delay of IVT depends on volume according to a convex function. Specifically, *b*_*j*, *IVT*_ is a function of the volume of the location $v := {\sum }_{i} w_{i} y_{ij}$; that is the IVT time *b*_*j*, *IVT*_ = *b*(*v*) is convex in the volume *v*. In our MILP model, we approximate the in-hospital delay of IVT by using *N* piece-wise linear functions, see Fig. 3 for an example with *N* = 3. These linear functions may be constructed from *b*(*v*) based on the tangent lines of *b*(*v*) in the points *v*_*n*_, *n* = 1,…,*N*. The *n* th tangent line $\hat b_{n}(\cdot )$ has the form $\hat b_{n} = \alpha _{n} \cdot v + {\upbeta }_{n}$, where the slope *α*_*n*_ and intersect β_*n*_ of the *n*-th linear function should be chosen such that $b(v_{n}) = \hat b_{n}(v_{n})$ and $b^{\prime }(v_{n}) = \alpha _{n}$ (Table 1).

**Table 1:** Notation for optimization models

**Sets**	
*I*	Set of demand locations (of suspected stroke).
*J*	Set of potential stroke centre locations.
*C*	Set of treatments types (*C* = {*IV* *T*, *IAT*}).
**Parameters**	
*P*(*IAT*)	Probability that patient requires IAT treatment.
*w*_*i*_	Demand at location *i*.
*d*_*ij*_	Travel time from location *i* to location *j*.
*r*_*c*_	Minimum number of patients with treatment *c*.
*p*_*c*_	Maximum number of stroke centres that provide treatment type *c*.
*b*_*j*, *IAT*_	In-hospital delay before start of IAT for stroke centre *j*.
**Decision variables**	
*y*_*ij*_	1 if demand point *i* is assigned to PSC *j* for IVT, 0 otherwise.
*v*_*ij*_	Flow of patients from PSC *i* to CSC *j* for IAT at location *j*.
*h*_*jc*_	1 if treatment of type *c* is given at location *j*, 0 otherwise.

As *b*_*j*, *IVT*_ depends on the volume and thus on the allocation protocol, we need an auxiliary variable *z*_*ij*_ representing the total in-hospital delay for IVT for patients from demand location *i* receiving IVT at PSC location *j*. We refer to Table 2 for notation related to the IVT in-hospital delay and to Table 1 for the rest of the notation.

**Table 2:** Notation for piece-wise linear approximation of IVT in-hospital delay

*α*_*n*_	Slope of the *n* th tangent line of IVT in-hospital delay.
β_*n*_	Intersect of the *n* th tangent line of IVT in-hospital delay.
*b*_*j*, *IVT*_	In-hospital delay for IVT at stroke centre *j*.
*z*_*ij*_	Total in-hospital delay for IVT for demand *i* in stroke centre *j*.

#### *Remark 4*

For acute stroke, we are primarily interested in scenario’s where in-hospital delays are a decreasing function of patient volume. In case patient volumes form a considerable part of the overall volume offered at an emergency department, large volumes may also lead to congestion, which may be characterized using queueing models [43–47]. It is well known that the mean waiting times of the traditional M/M/c and M/G/1 queues are a convex function of the offered load and thus the arrival rate (i.e. volume of patients). As such, the model design is also applicable to situations where congestion phenomena occur; this is not the case for stroke care in our setting as the average number of newly arriving stroke patients is at most two per day.

### MILP formulation

We now formulate the MILP for the optimal PSC and CSC locations and allocation protocol. The solution of the MILP provides the desired PSC and CSC locations (*h*_*j*, *IVT*_ and *h*_*j*, *IAT*_) and the corresponding optimal allocation protocol (*y*_*ij*_ and *v*_*ij*_). The objective is to minimize the sum of the SDST (Scene-Departure-to-Start-Treatment) over all patients in the region. The objective function, i.e., the total SDST, in (2) is divided in two parts. The first part is the total delay from scene departure until IVT. The second part is the total additional delay from IVT until start of IAT.

Now, let *M* be a sufficiently large number. The constraints (3) make sure that every demand point is assigned to a PSC. Constraints (4) regulate the allocation of patients from a PSC to a CSC in case IAT is required. In particular, it states that the flow of patients into PSC *j* that need IAT equals the flow out of PSC *j*. Constraints (5) and (6) guarantee that the minimum number of IVT and IAT patients are treated in case the location is open as a PSC and CSC, respectively. Similarly, constraints (7) and (8) provide that stroke centre *j* needs to be open for IVT and IAT, respectively, in case IVT and IAT patients are assigned to stroke centre *j*. Constraints (9) makes sure that the number of stroke centres of type *c* is less than or equal to *p*_*c*_.

2 $$ \begin{array}{@{}rcl@{}} \min Z_{opt} &=& \sum\limits_{i=1}^{|I|} \sum\limits_{j=1}^{|J|} \left[ y_{ij}w_{i}d_{ij}+z_{ij} \right] \\ &+& \sum\limits_{j=1}^{|J|}\sum\limits_{c=1}^{|J|} \left[ v_{jc}(d_{jc}+b_{c,IAT}) \right] \end{array} $$

3 $$ \text{s.t.} \qquad\sum\limits_{j=1}^{|J|} y_{ij}  =  1 \text{ } \forall i  $$

4 $$ \sum\limits_{i=1}^{|I|} y_{ij} w_{i} P(IAT)  =  \sum\limits_{c=1}^{|J|} v_{jc} \text{ } \forall j  $$

5 $$ \quad \sum\limits_{i=1}^{|I|} y_{ij} w_{i}  \geq  r_{IVT} h_{j, IVT} \text{ } \forall j  $$

6 $$ \quad\quad \sum\limits_{i=1}^{|I|} v_{ij}  \geq  r_{IAT} h_{j, IAT} \text{ } \forall j  $$

7 $$ \quad\quad \sum\limits_{i=1}^{|I|} y_{ij}  \leq  M h_{j, IVT} \text{ } \forall j  $$

8 $$ \quad\quad \sum\limits_{i=1}^{|I|}v_{ij}  \leq  M h_{j, IAT} \text{ } \forall j  $$

9 $$ \quad\quad \sum\limits_{j=1}^{|J|} h_{jc}  \leq  p_{c} \text{ } \forall c  $$

10 $$ \quad\quad z_{ij}  \leq  M y_{ij} \text{ } \forall i,j  $$

11 $$ \begin{array}{@{}rcl@{}} z_{ij}  &\leq&  b_{j, IVT} w_{i} \text{ } \forall i,j \end{array} $$

12 $$ \begin{array}{@{}rcl@{}} z_{ij}  &\geq&  b_{j, IVT} w_{i} - (1-y_{ij}) M \text{ } \forall i,j \end{array} $$

13 $$ \begin{array}{@{}rcl@{}} b_{j,IVT}  &\geq&  \sum\limits_{i=1}^{|I|} \alpha_{n} w_{i} y_{ij} + {\upbeta}_{n} \text{ } \forall n, i, j \end{array} $$

14 $$ \begin{array}{@{}rcl@{}} y_{ij}  &\in&  \{0,1\} \text{ } \forall i,j \end{array} $$

15 $$ \begin{array}{@{}rcl@{}} v_{jj^{\prime}}  &\ge&  0 \text{ } \forall j,j^{\prime} \end{array} $$

16 $$ \begin{array}{@{}rcl@{}} h_{jc}  &\in&  \{0,1\} \text{ } \forall j,c \end{array} $$

17 $$ \begin{array}{@{}rcl@{}} z_{ij}  &\ge&  0 \text{ } \forall i,j \end{array} $$

18 $$ \begin{array}{@{}rcl@{}} b_{j,IVT}  &\ge&  0 \text{ } \forall j \end{array} $$

Observe that the total in-hospital delay from the demand at location *j* is ${\sum }_{i} y_{ij} w_{i} b_{j,IVT}$, where the in-hospital delay per patient *b*_*j*, *IVT*_ depends on IVT volume at the corresponding location. Equations (10)–(12) avoid the multiplication of the variables *y*_*ij*_ and *b*_*j*, *IVT*_ by introducing the auxiliary variable *z*_*ij*_. Specifically, the equations make sure that *z*_*ij*_ = 0 if *y*_*ij*_ = 0 and *z*_*ij*_ = *w*_*i*_*b*_*j*, *IVT*_ if *y*_*ij*_ = 1. Constraints (13) model the in-hospital delay per patient at location *j* depending on total IVT volume at location *j* using its convex relationship (and approximate this function by *n* piece-wise linear functions). Finally, the constraints (14)–(18) guarantee that all decision variables are non-negative and that *y*_*ij*_ and *h*_*jc*_ are binary.

### Drip-and-ship and mothership

Although the allocation protocol is given in this section, an optimization model can be used to determine which treatment locations should be a CSC (and then also a PSC for drip-and-ship). In fact, using the optimization model it may be determined whether performance is improved when some stroke centres no longer act as a PSC and CSC. For the results, we do not take such scenario’s into account. Next, we first discuss the drip-and-ship protocol, followed by some comments regarding mothership.

The difference between the drip-and-ship protocol and the formulation in Section 4.2 is that we need to ensure that a patient is assigned to the nearest PSC. For any PSC *i*, let $J_{ij}^{*}$ be the set of PSCs that are closer to demand *i* than PSC *j* (which may also be an empty set). Then, in addition to all constraints in Section 4.2, we need to impose the following constraints

$$ \sum\limits_{k \in J_{ij}^{*}} h_{k,IVT}  \leq  (1-y_{ij})M \qquad\quad\quad\qquad\forall i,j $$

This constraint regulates that no PSC in the set $J_{ij}^{*}$ may be opened if demand *i* is assigned to PSC *j*, i.e., demand should be assigned to the nearest open PSC. *M* is again a sufficiently large number.

Observe that in the formulation above, some of the stroke centres may no longer provide IVT (and should then not be regarded as a stroke centre anymore). Although this improves the mean SDST, a typical practical implementation may be a scenario where all considered stroke centres should at least provide IVT. This can easily be accomplished in the model by modifying Constraints (9) into

19 $$ \sum\limits_{j=1}^{|J|} h_{jc}  =  p_{c} \qquad\qquad\qquad\qquad\qquad\forall c $$

combined with the appropriate choice of *p*_*c*_.

In case of the mothership protocol, the model reduces to a single-flow system as stroke centres can no longer be only PSCs. As such, the problem is actually a regular facility location problem. The key distinguishing feature, however, is that in-hospital delays still depend on patient volume. The formulation of the MILP for mothership can be found in Appendix A.

## Numerical experiments

In this section, we compare the different allocation protocols using six different regions that differ in the number of demand points, population size, surface area, demand distribution and the potential stroke centre locations. In Section 5.1 the experimental setup is explained. In Section 5.2 a single instance is highlighted for illustration. Finally, in Section 5.3 a comparison of the different allocation protocols is given, with the computational time given in Section 5.4.

### Regional structure and parameters

In this subsection, we first discuss the regional structures, followed by the choices of parameters.

We apply the models to 6 of the 24 ambulance regions (RAVs) in the Netherlands: Amsterdam-Amstelland, Holland-Midden, Haaglanden, Utrecht, Twente, and Groningen. The regions are chosen based on population density, following the classification in [38]; that is, we consider urban (Amsterdam & Haaglanden), rural (Groningen & Twente) and mixed (Holland-Midden & Utrecht) regions. As demand points, we take the four-digit postal codes.

Figure 10 in Appendix B shows the lay-out of the six regions. The surface area varies between 282 *km*^2^ (Amsterdam-Amstelland) and 2336 *km*^2^ (Groningen). Recall that the total demand in a region is equal to the model parameter *P*. We assume that every demand location is assigned a fraction of *P* proportional to the population density provided by the RIVM. For the potential PSC and CSC locations per region, we follow the categorization of [37]. In Fig. 10, the PSCs are indicated with a small circle and the CSCs with a cross.

Next to the regional structure, we use the parameters as indicated in Table 3. For each region, this provides 39 instances^1^ with a different parameter combination. The probability of IAT eligibility is based on expert opinion about the range of this probability [20]; in current clinical practice 20*%* is realistic, but this value is expected to increase. The minimum IVT requirement (*r*_*IVT*_) is set to ‘0’ to mimic current practice and avoid that the drip-and-ship protocol becomes infeasible due to the patient volume for one of the PSCs dropping below *r*_*IVT*_. Finally, the in-hospital delay for IVT is chosen according to the function in Fig. 3, ranging from 20 to 60 minutes, depending on patient volume. The in-hospital delay for IAT (*b*_*j*, *IAT*_) is 29 minutes.

**Table 3:** Parameter values for numerical experiments

Parameter	Experiment values
*P*(*IAT*)	{20*%*, 30*%*, 40*%*, 50*%*, 60*%*}
Total number of patients (*P*)	{300, 600, 900}
Minimum IAT requirement (*r*_*IAT*_)	{50, 100, 150}

The allocation protocols are compared based on the total delay from scene departure to start treatment (i.e., the SDST), corresponding to the objective function *Z* of the MILP. The relative difference in SDST for drip-and-ship and mothership, compared to the optimal protocol, is denoted by

$${\Delta}_{p} = \frac{Z_{p} - Z_{opt}}{Z_{opt}} \times 100\%,$$

where *Z*_*p*_ is the objective function for protocol *p* and *Z*_*opt*_ is the objective function of the optimal model.

### Insights from a single instance

To obtain insight in the impact of the different protocols, we first elaborate on a single instance. We focus on the Amsterdam-Amstelland region with *P*(*IAT*) = 20*%*, the total number of patients *P* = 600 per year and a minimum IAT requirement *r*_*IAT*_ of 50 per CSC.

The results of this Amsterdam-Amstelland instance is given in Table 4. The optimal allocation is better than drip-and-ship by 7.7% and than mothership by 11.5%. Moreover, the number of PSCs differs between the protocols; the optimal protocol has one less open PSC than drip-and-ship, whereas there are by definition no open PSCs for mothership. Finally, the fraction of patients that require IAT and need to be transferred is 90.3% for the drip-and-ship protocol; only 9.7% of the IAT patients are directly allocated to a CSC. For the optimal protocol, the percentage of required IAT transfers decreases slightly to 81.0%. We note that these high number of IAT transfers can be explained by the fact that the CSC is on the outskirts of Amsterdam and at the edge of the region.

**Table 4:** Results for single ‘Amsterdam-Amstelland’ instance (*P*(*IAT*) = 20*%*, *P* = 600, *r*_*IAT*_ = 50)

	Optimal	Mothership	Drip-and-ship
Sum of SDST	19069	21274	20531
Relative difference Δ	-	11.5%	7.7%
Number of (PSC, CSC)	(3,1)	(0,1)	(4,1)
Fraction of transferred IAT patients	81.0%	0%	90.3%

**Fig. 4:**
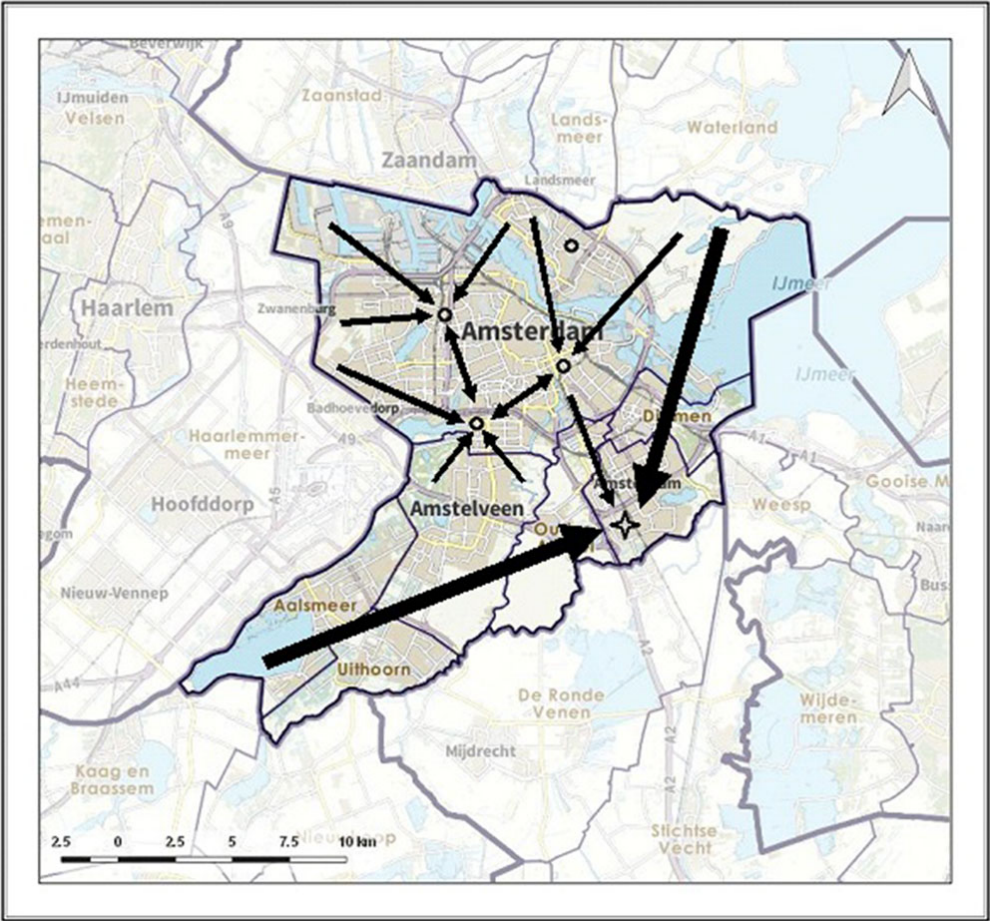
Allocation of demand according to optimal model

**Fig. 5:**
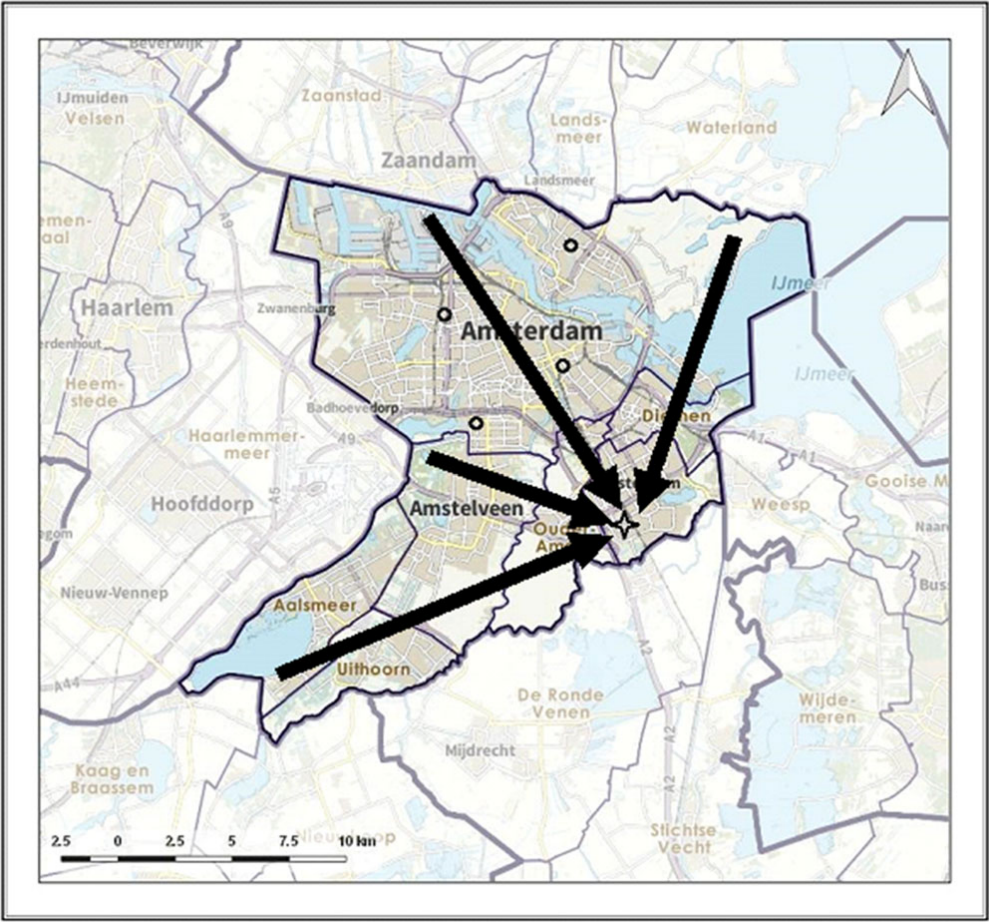
Allocation of demand according to mothership

**Fig. 6:**
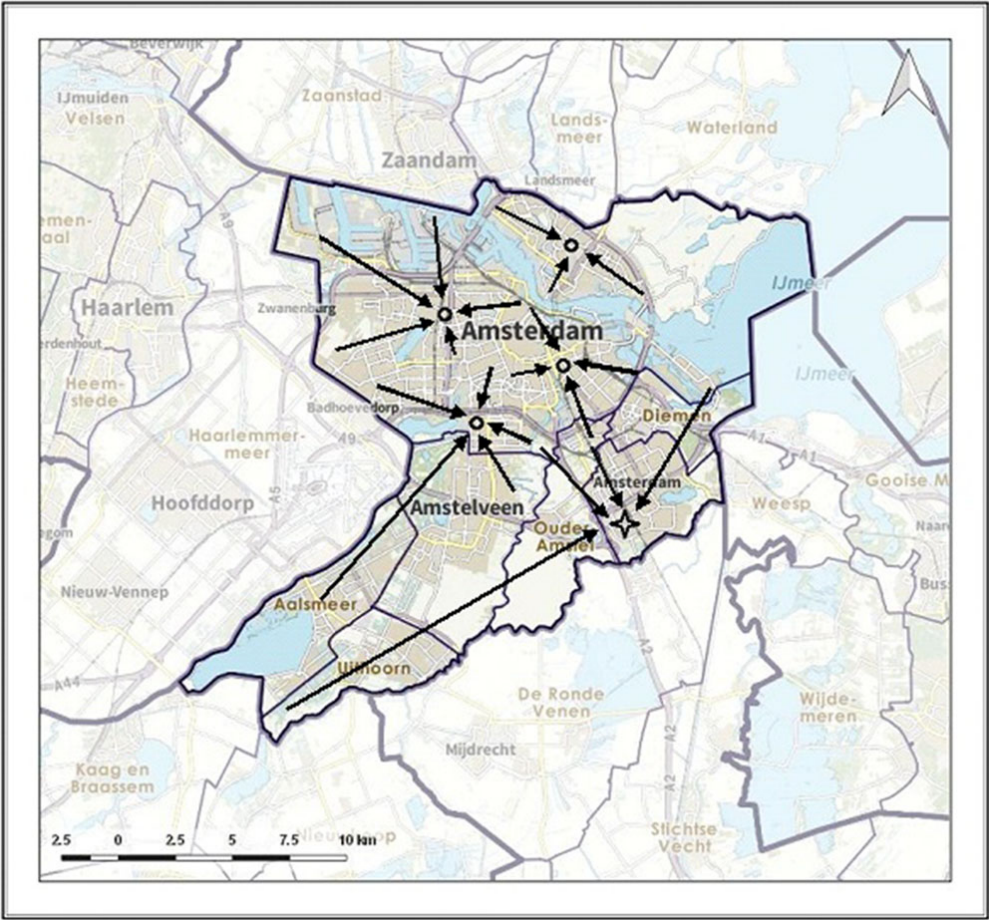
Allocation of demand according to drip-and-ship

In Figs. 4, 5 and 6 the allocation of demand to the stroke centres is indicated by black arrows. Figure 6 shows how patients are allocated to the nearest PSC, including the PSC at the top of the region, as required by the drip-and-ship protocol. Figure 4 shows that in the optimal protocol the PSC at the top of the region is no longer used for IVT patients. Also, for many locations, the optimal protocol allocates patients to the nearest PSC. An exception is the southeastern area, for which it is better to allocate patients directly to the CSC, which is similar to the allocation in the mothership protocol (as illustrated in Fig. 5).

For this single instance we observe that drip-and-ship outperforms mothership. This can be explained by the small percentage of patients that require IAT and the location of the CSC. Of course, results will strongly depend on the regional layout and health-related parameters.

### Analysis of different protocols

We compare the protocols for each region using the 39 feasible parameter combinations, resulting in a total of 234 instances.

Let us first focus on the difference between drip-and-ship and mothership. Table 5 shows the fraction of instances (in %) that mothership outperforms drip-and-ship in terms of SDST for the different regions and different values of *P*(*IAT*). Not surprisingly, and in line with Section 3, we see that mothership will perform better compared to drip-and-ship as *P*(*IAT*) increases. Next to *P*(*IAT*), the trade-off between the two practical protocols strongly depends on the region and the locations of stroke centres. For instance, we see that for the region Haaglanden mothership outperforms drip-and-ship for all instances. This can be explained by the degree of urbanization and corresponding short travel times. For the rural area Twente, we observe the opposite; only in some cases and for a *P*(*IAT*) of at least 50% it holds that mothership has shorter SDST than drip-and-ship. Roughly speaking, we can see that mothership gives better performance for urban areas, whereas drip-and-ship works well for rural areas, which can be explained by the impact of travel times.

**Table 5:** Fraction of instances (in %) in which mothership outperforms drip-and-ship per region for different values of *P*(*IAT*)

Region	Rurality	P(IAT)					
		20%	30%	40%	50%	60%	Total
Haaglanden	urban	100.0	100.0	100.0	100.0	100.0	100.0
Amsterdam	urban	16.7	14.3	62.5	66.7	100.0	56.4
Utrecht	mixed	50.0	57.1	100.0	100.0	100.0	84.6
Holland-Midden	mixed	0.0	0.0	0.0	0.0	100.0	23.1
Twente	rural	0.0	0.0	0.0	33.3	33.3	15.4
Groningen	rural	16.7	14.3	25.0	100.0	100.0	56.4

In addition, the location of the CSC(s) seem to play a crucial role. Amsterdam and Haaglanden are both urban areas, but for Amsterdam the CSC is located near the edge of the region. In Amsterdam, for smaller values of *P*(*IAT*), the longer travel times to a CSC in the mothership protocol do not outweigh the additional travel times due to transfers of IAT patients in drip-and-ship. Similar arguments apply to the other regions; the CSCs in Twente (urban) and Holland-Midden (mixed) are closer to the edge of the region compared to Groningen (urban) and Utrecht (mixed).

**Fig. 7:**
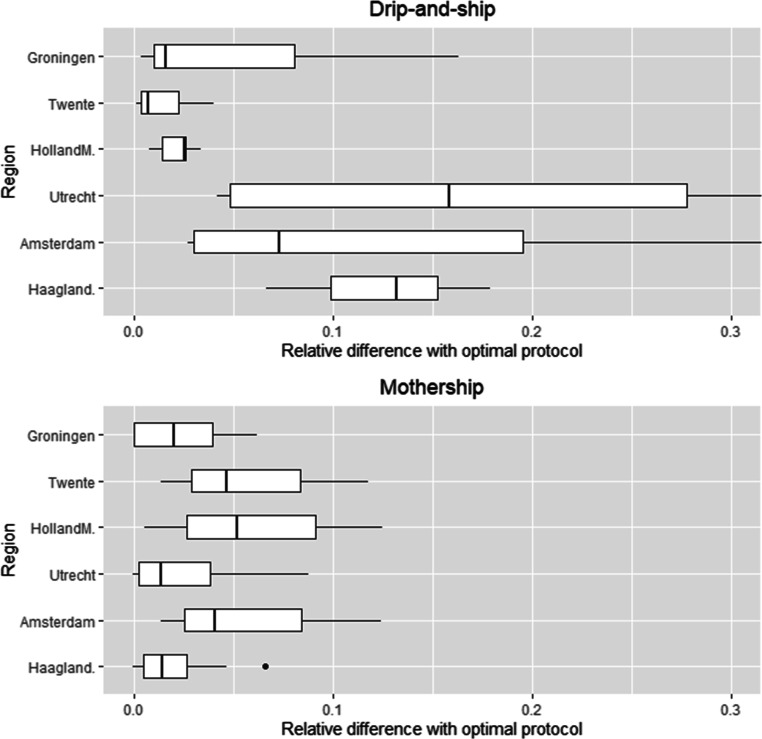
Relative differences with optimal protocol for drip-and-ship (top) and mothership (bottom)

In Fig. 7 the relative differences (Δ_*p*_) between drip-and-ship (left) and mothership (right) compared to the optimal protocol are visualized using boxplots based on the 39 instances per region. The observations above concerning Table 5 also apply to a large extent to the performance per region in Fig. 7. For instance, we see again that overall mothership performs better than drip-and-ship in the urban regions, where the reverse holds for rural areas (except for some cases with *P*(*IAT*) ≥ 50*%*). The variability in performance for drip-and-ship can be considerably larger than for mothership. For example, for the regions Amsterdam and Utrecht the performance of drip-and-ship is almost 40% worse than optimal for *P*(*IAT*) = 60*%* and *P* = 300. For mothership, the performance is only roughly 12% worse than optimal for the regions Amsterdam, Holland-Midden, and Twente in case *P*(*IAT*) = 20*%* and *P* ≥ 600. On average the total SDST when using the drip-and-ship protocol is 8.6% larger than the optimal model, whereas for the mothership protocol this is only 3.9%. Nevertheless, the mothership protocol can perform worse than the drip-and-ship protocol in all regions (except Haaglanden). Specifically, for the regions Holland-Midden, Twente and Groningen, the mothership protocol performs worse in 68.4% of the instances. Over all instances, the fraction of IAT patients that need to be transferred from PSC to CSC in the optimal model is 59.4% on average with a standard deviation of 25.1%. This shows that the required number of transfers will be considerable, but strongly depends on the region and health-related parameters.

#### Sensitivity of parameters

**Fig. 8:**
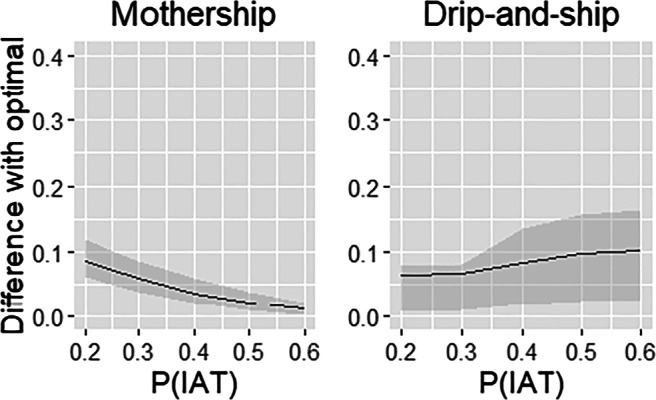
Mean relative difference with the optimal protocol against *P*(*IAT*) (the area between the 25% and 75% percentile is shaded)

**Fig. 9:**
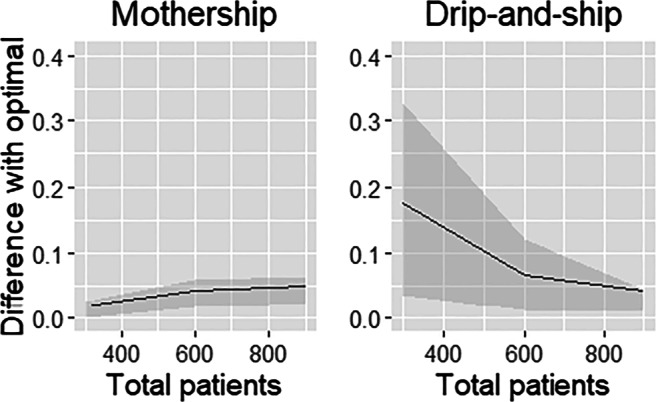
Mean relative difference with the optimal protocol against *P* (the area between the 25% and 75% percentile is shaded)

**Fig. 10:**
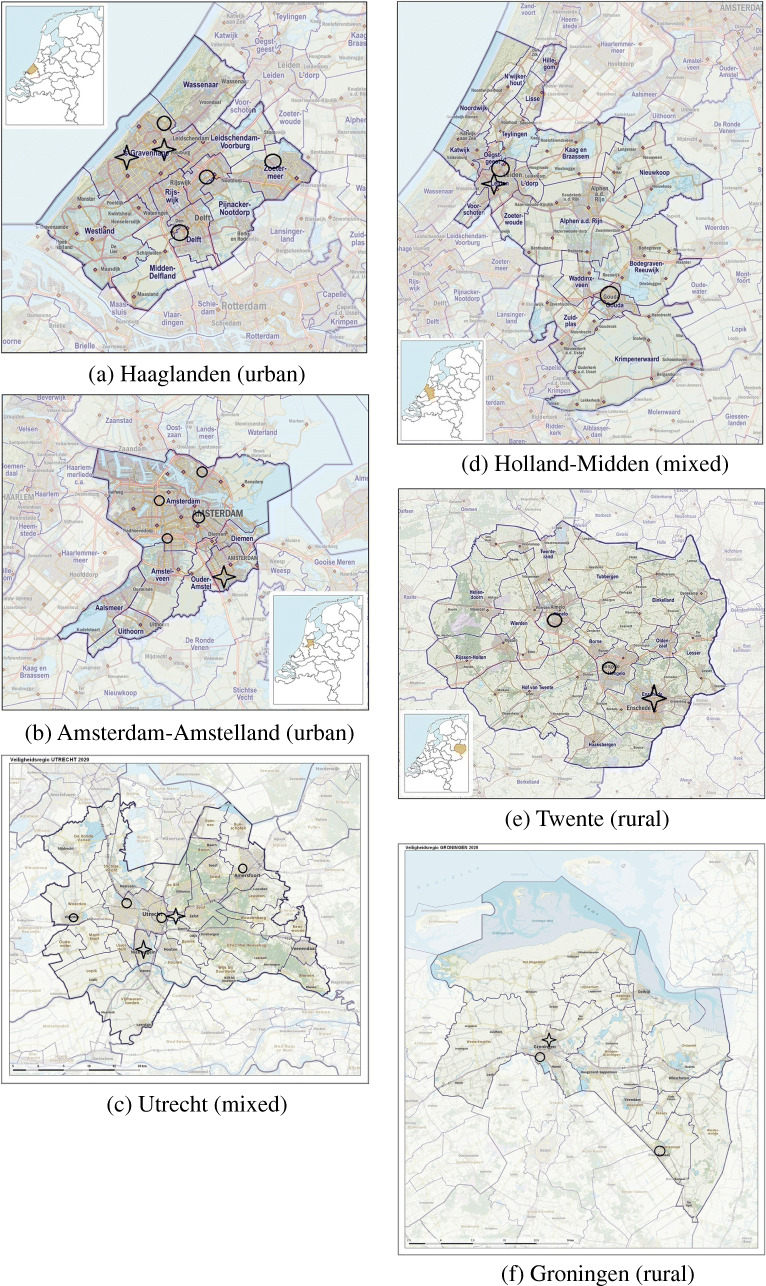
Lay-out of the six regions

So far, we primarily focused on the impact of the regions on the performance. Below, we further explore the impact of *P*(*IAT*) and the total demand *P*; the impact of the minimum IAT requirement *r*_*IAT*_ is related to the impact of *P*(*IAT*) and *P*. In Figs. 8 and 9 you may find the mean (line) and interquartile range (shaded area) of the total SDST for the two practical protocols relative to the optimal protocol against *P*(*IAT*) and *P*, respectively. As may be expected, the performance of the drip-and-ship protocol decreases as *P*(*IAT*) increases, whereas the performance of mothership improves. In fact, for *P*(*IAT*) = 60*%* we see that SDST for mothership is close to optimal. For small values of *P*(*IAT*), e.g. when *P*(*IAT*) = 20*%*, drip-and-ship typically performs better than mothership, but the performance is not necessarily close to optimal. Specifically, in the case with small patient volumes (*P* = 300), the SDST of drip-and-ship for Utrecht is still 41.9% worse than optimal.

Figure 9 shows the performance of the protocols relative to optimal as the total number of patients *P* varies. For *P* = 300, the performance of drip-and-ship varies considerably. Specifically, the performance for the regions Utrecht and Amsterdam is quite poor in that case, and are at least 37% (Utrecht) and 31% (Amsterdam) worse than optimal for all values of *P*(*IAT*). This can be explained by the in-hospital delay that becomes rather big when there are quite some stroke centres (6 and 5 for Utrecht and Amsterdam, respectively) and the total patient volume is small. These examples show that drip-and-ship is vulnerable when the number of patients per stroke centre becomes small; the order of magnitude also clearly depends on the function chosen for *b*_*IVT*_ when patient volumes become small. For mothership, the relative performance compared to optimal decreases when *P* increases. Again, this follows from in-hospitals delay; as *P* is larger, the optimal protocol will use more PSCs for its allocation without excessive in-hospital delays for IVT.

#### *Remark 5*

Although the drip-and-ship and mothership protocols may show close to optimal performance in some instances, it seems difficult to give any reasonable performance guarantees. For instance, consider a simplified situation with *P*(*IAT*) = 0, such that we may focus on IVT. Assume that there are 3 demand locations (*I* = 3) and two possible PSC locations (*J* = 2) that are the same as demand locations 1 and 2. Moreover, let *d*_12_ = *d*_21_ = *D*, *d*_31_ = *D* − *δ* and *d*_32_ = *δ* for some *δ* > 0 sufficiently small and *D* large. Consider the following demands: *w*_1_ = *r*_*IVT*_ − *𝜖*, *w*_2_ = *r*_*IVT*_, and *w*_3_ = *𝜖* for some small *𝜖* > 0.

In the optimal allocation, both PSC locations are open and patients of locations 1 and 3 are assigned to PSC location 1 such that the minimum volumes of IVT are met for both PSCs. The optimal total travel time is then *𝜖*(*D* − *δ*), which is only due to patients from location 3. When applying a drip-and-ship protocol it is not possible to open both locations, as PSC location 2 would be the nearest PSC to demand location 3. Hence, for drip-and-ship it is only feasible to open one location without violating the minimum volume requirements. Opening location 2 as a PSC, then provides the smallest total travel time of (*r*_*IVT*_ − *𝜖*) × *D* + *𝜖* × *δ* = *r*_*IVT*_*D* − *𝜖*(*D* − *δ*). Observe that both the absolute and relative difference in total travel time between optimal and drip-and-ship explode when $D \to \infty $ and *𝜖* *↓* 0.

### Computational time

The numerical experiment is run on an Intel Core i7-4770k CPU @ 3.50GHz with 24 GB RAM. In this experiment, a single instance of the model takes approximately one minutes; with 270 instances and three models (mothership, drip-and-ship, and optimal) the total experiment takes roughly 13 hours to complete.

## Conclusion and discussion

In this paper, we model the joint decision of PSC and CSC locations and the allocation of acute stroke patients in a region as a two-level hierarchical facility location problem. Specifically, we include the impact of the volume of IVT patients on in-hospital delays in our MILP, and compare the optimal model with the protocols in practice: mothership and drip-and-ship.

From our numerical experiments we see that the performance of the protocols depends on a variety of elements. In general, mothership performs better than drip-and-ship in urban areas, in particular in case of more centrally located CSCs; vice versa, drip-and-ship performs well in rural areas and stroke centres spread over the region. Additionally, for *P*(*IAT*) ≤ 30*%* drip-and-ship seems preferable, unless the total patient volume per PSC becomes rather small, whereas mothership performs excellent for *P*(*IAT*) ≥ 50*%*. On average, mothership and drip-and-ship are 3.9% and 8.6% worse than optimal, whereas drip-and-ship seems more sensitive to parameter choices.

In our experiments, the optimal protocol leads to a decrease between 0 and 41.9 percentage points in SDST for a region. This corresponds to a gain between 0 and 18.9 minutes per treated patient, based on an average SDST of 45 minutes. A measure to evaluate the impact of medical treatments in economical terms is QALY’s (quality-adjusted life years) [33]. Within the first six hours of onset, every hour of delay results in an average loss of 0.77 QALY’s for IAT patients [36]. Assuming linearity, this results in an improvement between 0 and 0.24 QALY’s for an individual IAT patient. For a region with 150 IAT patients per year, with a value of € 50,000 per QALY [35], the value of medical intervention in acute stroke care has the potential to gain a value up to € 1,800,000 per year using the optimal model, although the potential strongly depends on the region and the parameters.

The model precisely prescribes which stroke centres should become PSCs and CSCs and how patients should be alloca ted depending on the location of onset. As a consequence, the flow of stroke patients, including patient transfers, will typically change and stroke centres will be faced with different patient volumes. As patient volume is key to any hospital, the potentially required modifications will be a subject of debate. Due to the rather recent introduction of IAT, there is now a practical need to re-establish the allocation of ischemic stroke patients. The insights from the numerical experiments provide an initial estimate of a proper organization and the potential gain. Moreover, the application of the optimal model for a specific region and situation should facilitate the debate about locations of PSCs and CSCs and the corresponding allocation. Even if the optimal model is not fully implemented, it shows the direction to look for when reorganizing acute stroke care.

Finally, there are some limitations to the model that may be studied in the future. The regional layout and the parameters, such as *P*(*IAT*) and *b*_*IVT*_, may have a considerable impact on which protocol is better, but these parameters are difficult to estimate in current practice. Also, the value of *P*(*IAT*) may change in the future and may depend on the population demographics and IAT eligibility criteria.

The overall observations from our numerical experiments provide insight in the differences between the two practical protocols, but for any region it would be preferred to apply an optimization for finding the actual optimal allocation.

Also, in the current model we minimize the total time SDST, but the actual criterion is the condition of the patient after IVT and/or IAT. From a computational perspective, it may be observed that the problem is NP-hard. This implies that the size of the region that can be calculated to optimality using an MILP formulation is limited.

## Appendix A : Mothership model

In this section, we provide the MILP formulation for the mothership protocol, i.e., the locations of CSC facilities. Since the allocation between stroke centres is no longer possible, Constraints (4) should be removed and the problem is now a single-level system. Due to the absence of PSCs, the notations may be simplified. However, we choose to use the same notation for consistency with the models for the other protocols.

Observe that Constraints (25) makes sure that each location either provides both IVT and IAT, or no treatment of acute stroke. Constraints (30) are similar to the drip-and-ship protocol and guarantees that each patient is allocated to the nearest location.

20 $$ \begin{array}{@{}rcl@{}} \text{Minimize} Z_{moth} &=& \sum\limits_{i=1}^{|I|} \sum\limits_{j=1}^{|J|} z_{ij} + y_{ij}w_{i}\\(d_{ij}&+&b_{j,IAT}P(IAT))\end{array} $$

21 $$ \text{s.t.} \sum\limits_{j=1}^{|J|} y_{ij}  =  1, \text{ } \forall i $$

22 $$ \sum\limits_{i=1}^{|I|} y_{ij} w_{i} \geq  r_{IVT} h_{j, IVT} \text{ } \forall j $$

23 $$ \sum\limits_{i=1}^{|I|} y_{ij} w_{i} P(IAT)  \geq  r_{IAT} h_{j, IAT} \text{ } \forall j $$

24 $$ \sum\limits_{i=1}^{|I|} y_{ij}  \leq  M h_{j, IVT} \text{ } \forall j $$

25 $$ h_{j, IVT}  =  h_{j, IAT} \text{ } \forall j $$

26 $$ z_{ij}  \leq  M y_{ij} \text{ } \forall i,j $$

27 $$ z_{ij}  \leq  b_{j, IVT} w_{i} \text{ } \forall i,j $$

28 $$ z_{ij}  \geq  b_{j, IVT} w_{i} - (1-y_{ij}) M \text{ } \forall i,j $$

29 $$ b_{j,IVT}  \geq  \sum\limits_{i=1}^{|I|} \alpha_{n} w_{i} y_{ij} + \beta_{n} \text{ } \forall n, i, j $$

30 $$ \sum\limits_{k \in J_{ij}^{*}} h_{k,IVT}  \leq  (1-y_{ij})M \text{ } \forall i,j $$

31 $$ y_{ij}  \in  \{0,1\} \text{ } \forall i,j $$

32 $$ h_{jc}  \in  \{0,1\} \text{ } \forall j,c $$

33 $$ z_{ij}  \ge  0 \text{ } \forall i,j $$

34 $$ b_{j,IVT}  \ge  0 \text{ } \forall j $$

## Footnotes

1 We only consider parameter combinations leading to feasible instances. For instance, with *P*(*I**A**T*) = 20*%* and *P* = 300, there are only 60 patients requiring IAT, such that *r*_*I**A**T*_ > 60 is not feasible. Consequently, we disregard six parameter combinations per region.
